# Considerations in Alzheimer’s Disease in Women

**DOI:** 10.1007/s11910-026-01504-3

**Published:** 2026-07-23

**Authors:** Caroline Just, Kaitlin Seibert, Carol Chan, Carolina Maldonado-Correa, Danita Jones

**Affiliations:** 1 Center for General Neurology, Cleveland Clinic, Cleveland Clinic Lerner School of Medicine at Case Western Reserve University, 9500 Euclid Ave. Mail Code S90, Cleveland, OH 44195 USA; 2 Center for Brain Health, Cleveland Clinic, Cleveland, OH USA; 3 Department of Neurology, Cleveland Clinic Florida, Weston, FL USA

**Keywords:** Alzheimer’s disease, Sex differences, Women, Tau PET, p-tau217, Cognitive reserve, Diagnostic delay, Atypical Alzheimer’s disease, Biomarkers, Menopause

## Abstract

**Purpose of Review:**

To examine sex differences in Alzheimer’s disease and cognition with a focus on hormonal transitions, biomarker trajectory, and implications for diagnosis and treatment.

**Recent Findings:**

Women account for nearly two-thirds of individuals with Alzheimer’s disease and demonstrate important biological and clinical differences compared with men. APOE ε4 confers greater risk in women, while menopause, depression, chronic stress, adverse pregnancy outcomes, and metabolic dysfunction may further increase vulnerability. Biomarker studies suggest that amyloid trajectories are broadly similar between sexes, but women exhibit earlier or greater tau accumulation once amyloid pathology is present. Women may maintain verbal memory performance longer than men despite underlying pathology, potentially delaying diagnosis. Emerging plasma biomarkers, particularly p-tau217, may improve early detection, monitoring, and treatment.

**Summary:**

Alzheimer’s disease in women reflects a complex interaction between sex-specific biology, hormonal transitions, psychosocial factors, and neurodegenerative processes. Recognizing these differences has important implications for cognitive assessment, biomarker interpretation, diagnosis, and application of disease-modifying therapies.

## Introduction

There are 6 million people living with Alzheimer’s Disease (AD) in the United States. Almost two thirds of those people – or nearly 4 million people – are women. Even after taking into account that women, on average, live longer than men, the risk for women to develop AD is much higher than the risk for men [1]. It is imperative that we understand the reasons for this difference, so that we can tailor screening, diagnostic testing, and treatments to specific populations [2]. This article will review the latest information on how genetics, neuropathology, pregnancy & menopause, and metabolic risk factors alter Alzheimer’s risk in women, and detection methods, patterns of atrophy, and protein deposition affect the disease trajectory differently in women.

### Genetics

The strongest risk factor for Alzheimer’s Disease is ApoE genotype. Possession of the ApoE4 allele is associated with a greater burden of beta amyloid deposition. Women who have one copy of ApoE4 are at greater risk of Alzheimer’s than men with one copy, and this risk is equivalent to that of men who have two copies of ApoE4. ApoE4 in women is also associated with a greater accumulation of phosphorylated tau [3–5].

## Insulin Resistance and Diabetes

Diabetes, similar to other vascular risk factors, is driven by the presence of insulin resistance. There has been evidence to suggest insulin can affect hippocampal-mediated processing due to CNS insulin receptors found in the hippocampus and surrounding limbic structures [6]. When comparing patients with MCI or Alzheimer’s Disease, insulin resistance is associated with more severe cognitive impairment independent of amyloid or tau accumulation [7]. Estrogens initially serves as a protector for cardiometabolic disorders in women. In perimenopausal and postmenopausal women, when estrogen levels are significantly reduced, the risk of insulin resistance increases [8]. Hormone therapy may become more important in the discussion of insulin resistance and potential increased risk of Alzheimer’s Disease specifically within the female population.

### Hypertensive Disorders of Pregnancy

Hypertensive Disorders of Pregnancy (HDP) include including preeclampsia, eclampsia, HELLP (hemolysis, elevated liver enzymes, and low platelet count) syndrome, gestational hypertension, and chronic hypertension.

A 2022 systematic review and meta-analysis of 183,874 women with and 2,309,705 women without HDP by Schliep et al. found a significant association between HDP and dementia. Women with HDP were 40% more likely to develop Alzheimer’s Disease, and 3x as likely to develop vascular dementia. A 2025 meta-analysis of 6,263,431 participants by Miller et al. found that women who had an adverse pregnancy outcome, defined as any of hypertensive disorders of pregnancy, gestational diabetes, stillbirth, fetal growth restriction, preterm birth, or placental abruption, were associated were a higher risk of all-cause dementia, AD, and vascular dementia. Notably, when only hypertensive disorders of pregnancy were included as an adverse outcome, there was no longer a significant association with AD, though the association with all-cause dementia and vascular dementia remained. This suggests a complex relationship between HDP, other, adverse outcomes of pregnancy, and Alzheimer’s, and represents an area of current and future study [9].

### Menopause and Neurobiological Change

Estrogen receptors in the brain are highly expressed in brain areas involved in emotions and cognition, such as the amygdala and hippocampus. Estrogen is neuroprotective in a variety of ways, including increasing synaptic plasticity, dendritic spine density, and brain-derived neurotrophic factor [10]. Estrogen plays a role in cleaving amyloid precursor protein to a non-plaque state [11, 12]. The role of progesterone on cognition is less clear, although a protective effect in suggested [12]. When menopause occurs, the ovaries no longer produce estradiol, and instead estrone, the main estrogen in the female body after menopause, is produced primarily in adipose tissue. Estrone is much less potent than estradiol. Brain-derived estrogen is estradiol, but the overall effect of menopause is a significant reduction in estrogens in the body. It is postulated that the rapid decrease in estrogen levels that occurs with menopause increases the deposition of amyloid plaques by removing the protective effect of estrogen on amyloid precursor processing [13]. The rapid drop in estrogen worsen depressive symptoms and impair memory and recall [12]. Regardless of estrogen’s role in vivo pre-menopause, treatment with menopause hormone therapy (MHT) does not reduce risk of dementia [14].

Objectively measured vasomotor symptoms correlate with poorer cognitive performance, but self-reported symptoms do not. Sleep disturbances, including insomnia and sleep-disordered breathing during and after perimenopause may also negatively impact cognition [15, 16]. Poor sleep quality in recently menopausal women has been shown to be associated with an increase in amyloid deposition, but not consistently so [17]. Younger age at surgical menopause is associated with cognitive decline and Alzheimer pathology in older women [18, 19]. Volume of hot flashes during sleep correlated with number of white matter lesions in perimenopausal women without other clinical evidence of vascular disease.

### Depression

Depression is a risk factor for Alzheimer’s and all-cause dementia [20]. Although late-life depression has the strongest risk, depression in earlier stages of life has also been associated with an increased risk dementia [21, 22]. While depression appears to confer greater risks of dementia in males, females are approximately twice as likely to experience major depression across their lifespan making it a significant contributor to dementia risk in females at a population level [22–25]. Female risk for depression is highest during reproductive years [24]. It remains prevalent throughout the perimenopausal period, and together with vasomotor symptoms and sleep disturbances can increase cardiovascular risk in women that later affect cognitive function [26]. For individuals with depression, timely access to effective treatments may mitigate the risk of progression to dementia, though more research in this area is needed.

### Chronic Stress

Chronic stress is associated with a 60% increased risk of dementia, with a dose-response relationship based on chronicity [27]. Chronic stress and depression together can have an additive effect on one’s risk of dementia [28]. The relationship between chronic stress and dementia is thought to be related to the chronic activation of the hypothalamic-pituitary-adrenal axis, resulting in prolonged, elevated levels of cortisol, which crosses the blood-brain barrier to affect key memory structures including the amygdala, hippocampus, prefrontal cortex [29]. Persistent inflammatory responses in the brain involving microglia and astrocytic activation may also have a role in accelerating neurodegenerative processes [30]. Animal models have further demonstrated that chronic stress may accelerate the deposition of amyloid plaques and hyperphosphorylation of tau, pathological hallmarks of Alzheimer’s disease [31].

Females experience multiple risk factors that contribute to chronic stress. Lower socioeconomic status is associated with greater risk of dementia and accelerated cognitive decline [32–34]. While most studies have focused on socioeconomic status in older adults, lower socioeconomic status even in childhood and early adulthood has been associated with worse memory function and cognitive decline [35]. Food insecurity in early life, midlife, and later adulthood have also been associated with increased risk of dementia [36, 37]. Across most countries, including the United States, women are more likely to experience poverty and food insecurity than men due to the gender wage gap, historically lower education levels, and increased likelihood of heading single-parent homes [38, 39]. Recent research has found that women may be more vulnerable than men to the effects of socioeconomic status on dementia risk despite having stronger interpersonal support, further augmenting its effects at a population level [40].

In addition to chronic stress from economic factors, women may face additional challenges due to discrimination and social roles. Individuals reporting more experiences of discrimination throughout their lifetime have a greater risk of dementia, independent of sociodemographic, clinical, and behavior risk factors [41]. Social roles such as caregiving, which is disproportionately carried out by women are also a significant risk factor for cognitive decline due to chronic stress, social isolation, and neglect of personal health [42].

## Neuropsychological Profile Differences in Women Versus Men with Alzheimer’s Disease

Verbal function tends to be higher in women than in men. In a recent study, cognitively normal women outperformed men on the ADAS Cog 13, particularly in verbal domains such as word recall, delayed recall, naming, and recognition; however, these sex differences largely diminish in Alzheimer’s disease. Greater hippocampal integrity was strongly associated with better cognitive performance across participants, and women’s early cognitive advantage, independent of hippocampal integrity, may mask initial decline and delay diagnosis [43].

Ryan et al. investigated cognitive performance in men and women with Alzheimer’s disease who had comparable levels of overall impairment and found that men performed better on tasks involving immediate prose memory, semantic fluency, semantic memory, and confrontation naming, whereas no differences were observed in delayed memory or general verbal fluency, and after adjusting for age, education, and disease duration, only the advantages in semantic fluency, semantic memory, and naming remained, suggesting that women show relatively greater deficits in semantic and language-related functions despite similar global cognition [44].

Levine et al. (2026) showed that women had better verbal learning and memory performance at baseline in both non-Hispanic White and Hispanic/Latino cohorts, and over time men exhibited greater decline, with cognitive deterioration more evident in non-Hispanic White men and worsening clinical symptoms in Hispanic/Latino men, while overall patterns of decline were similar across ethnic groups and were not influenced by Alzheimer’s disease biomarkers [45].

### Cognitive Reserve and Verbal Memory

In a study by Emrani et al., sex/gender differences in Alzheimer’s disease were observed not only in prevalence but also in diagnostic accuracy and clinical progression, with implications for early detection. These differences varied by stage, with females showing an advantage in verbal memory preclinically but a faster decline during mild cognitive impairment. This pattern may be partly explained by a lifelong female advantage in verbal memory, which can mask early deficits in a domain central to diagnosis [46].

This advantage is frequently interpreted as a form of cognitive reserve, enabling women to maintain cognitive performance despite underlying brain changes. In this study it was found that in the Rey Auditory Verbal Learning Test (RAVLT), women outperform men in early stages but decline more sharply as Alzheimer’s progresses, and a significant sex-moderation effect was observed in the right isthmus cingulate, where greater cortical thickness is associated with better verbal learning in women, particularly in those with amyloid positivity [47].

As Alzheimer’s disease pathology increases, particularly with rising β-amyloid levels, this advantage diminishes and may result in more rapid cognitive decline once compensatory mechanisms are exceeded [48].

### Detection of Cognitive Impairment in Highly Educated Women

Low scores on neuropsychological tests are commonly used as objective indicators of cognitive impairment; however, this approach may underestimate deficits in high-functioning individuals, who are less likely to obtain low scores, thereby requiring more sensitive methods that also consider the absence of high performance [49]. Higher educational attainment is associated with better cognitive performance and a reduced risk of cognitive decline, which may contribute to masking early deficits and delaying their clinical detection. This was demonstrated in a large cohort of community-dwelling older women in which multiple cognitive domains were assessed longitudinally, and higher levels of education were consistently linked to better baseline performance and less subsequent decline [50]. Older adults with higher education levels generally have a lower risk of developing dementia, often due to cognitive reserve, which can delay clinical symptoms. However, cognitive reserve may hinder early detection, as individuals can perform normally on standard assessments even with mild cognitive changes. Elkana et al. examined this challenge in a longitudinal study using structural MRI and a comprehensive set of neuropsychological tests. The study included highly educated, cognitively active older adults with above-average intellectual functioning. Results indicated that increasing age was linked to reduced grey matter and overall brain volume, along with ventricular enlargement. Investigators also ound that the RAVLT, semantic verbal fluency, Rey Osterrieth Complex Figure copy, and MoCA were more sensitive to early decline, showing significant reductions over 12 months. In contrast, phonemic fluency, the Trail Making Test, and digit span did not show significant changes. These findings highlight the importance of using sensitive measures and longitudinal assessment to detect subtle cognitive decline, and support the need for norms that account for both age and education [51].

## Subjective Cognitive Decline (SCD)

Subjective Cognitive Decline (SCD) refers to self-perceived worsening of cognitive function despite normal performance on objective tests and has been identified as an early marker of neurodegenerative processes. Individuals with SCD have an increased risk of developing mild cognitive impairment (MCI), Alzheimer’s disease, and dementia, with symptoms often preceding clinical diagnosis by several years [52].

Higher amyloid burden has been associated with more severe subjective cognitive complaints across multiple domains. Although women may exhibit higher amyloid levels, they do not necessarily report more severe complaints, suggesting possible sex differences in symptom perception and reporting [53].

## Neuropathology

**Fig. 1:**
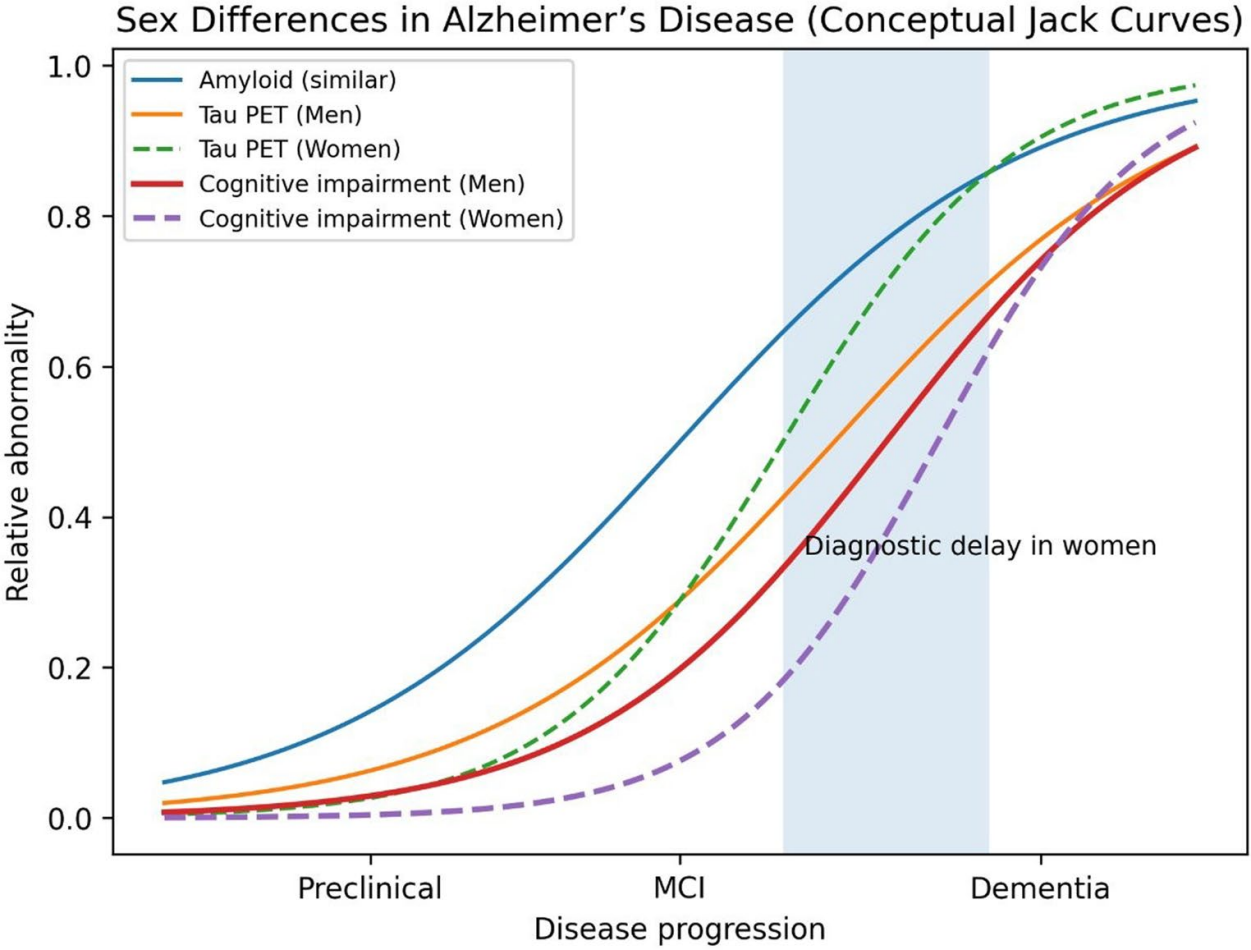
Simplified sex-specific Alzheimer disease trajectories. Amyloid accumulation is broadly similar in women and men, whereas women show earlier or greater downstream tau burden and delayed but steeper clinical impairment, contributing to diagnostic delay

The hallmark of diagnosis for Alzheimer’s Disease involves the deposition of amyloid-β and tau into the medial temporal lobe, with progression to the remaining neocortex over time. The total burden of amyloid plaque increases for one to two decades during the preclinical phase of Alzheimer’s Disease. This is contrast to neurofibrillary tangles, measured by tau PET, that develop later and increase with symptom severity. Women and men appear to share broadly similar amyloid trajectories in Alzheimer disease, with some differences discussed below. In contrast, women more often show earlier or greater tau burden once amyloid is present and can remain clinically comparable for longer because of domain-specific cognitive reserve, especially verbal memory. This combination can delay diagnosis in women, then be followed by a steeper clinical decline, supporting sex-aware assessment, biomarker interpretation, and earlier etiologic confirmation when symptoms are subtle. Figure 1 provides a simplified sex-specific Jack-curve model for Alzheimer disease. The teaching point is not that women and men follow different diseases, but that they move through the same cascade with different timing and clinical expression. Across cohorts, amyloid accumulation appears more similar by sex than tau accumulation. By contrast, women more consistently show greater tau burden, faster tau accumulation, or earlier tau positivity once amyloid is present [54–60].

Women show a differential tau response to amyloid-β and subsequent p-tau secretion [61]. In addition, females show a faster rate of decline after diagnosis of MCI or Alzheimer’s Disease even in the setting of similar PET Amyloid centiloid values and CSF analysis. Yet even in cognitively intact individuals, the female sex has consistently demonstrated more amyloid-β plaque as well as more widespread tau pathology. This has been correlated with tau-PET imaging. This raises the question of women having a higher resilience against low levels of tau pathology clinically, allowing females to remain cognitively intact when compared to male cohorts with similar tau levels. Tau PET findings, however, require methodological caution. Apparent cortical sex differences can be exaggerated by higher meningeal and skull off-target binding in women for several tracers, so strong claims should ideally be robust to surface-based analyses, exclusion of regions near meninges, and partial-volume correction. Even with that caveat, the weight of current evidence still favors a real biological divergence in downstream tau burden rather than a purely imaging artifact [54–59, 61].

In the serum, baseline p-tau181 levels may be similar in men and women, yet the downstream meaning of an elevated result can differ. In Alzheimer’s Disease Neuroimaging Initiative (ADNI) sex modified the association of plasma p-tau181 with amyloid PET, tau PET, FDG metabolism, cognitive decline, and conversion risk, suggesting that a borderline biomarker value in a woman may signal nearer-term clinical change than the same value in a man. A useful synthesis is a two-stage model: women appear to show stronger amyloid-to-tau coupling, whereas men may show steeper neurodegenerative or cognitive consequences per unit tau in some clinic settings. The practical implication is that amyloid is usually the weaker discriminator of sex differences, whereas tau is the more informative marker for prognosis and clinical interpretation [54–59, 61, 62]. With higher p-tau217 levels, females show a greater rate of cognitive decline despite prior baseline levels of cognitive performance. Monitoring levels of plasma p-tau217 levels may allow for earlier detection, disease monitoring, and treatment decision-making [63].

Within the aging population, women tend to have better verbal recall memory than respective age matched male counterparts, which possibly contributes to a later diagnosis and subsequent presumed faster rate of decline [2]. Females have also been shown to have a higher rate of tau accumulation within the entorhinal cortex compared to males. It may be the secretion of phosphorylated tau (p-tau) that is the key role between the formation of amyloid plaques and the aggregation of tau. Levels of p-tau are affected directly by amyloid-β levels. This increased level of tau was, in turn, associated with a higher level of amyloid-β plaques. In a study looking at tau aggregation differences between sexes, it was found that women had a significantly higher baseline p-tau217 levels at higher amyloid β centiloid values compared with men [54]. For each increase in Aβ concentration, there is an associated increase of p-tau217 level by between 0.96% and 1.64% in females compared with an increase of p-tau217 level by 0.62% to 1.30% in men [54]. Subsequently, each additional unit of Alzheimer’s pathology is associated with a 22x increased odds of Alzheimer’s Disease in women, compared to only a 3x increased odds in men [2] .

Females show faster tau accumulation in several regions of the brain, including the superior and inferior parietal cortex, rostral middle frontal gyrus, precuneus cortex, fusiform gyrus and inferior temporal gyrus [54]. MRI shows annual atrophy rates are faster in women when compared to men, regardless of cognitive integrity. Baseline structural differences between males and females, including greater cortical thickness in females across multiple brain regions in cognitively normal older adults, progressively diminish as Alzheimer’s disease advances. The number of regions showing significant sex differences decreases in mild cognitive impairment and disappears entirely in established Alzheimer’s dementia, indicating that the neurodegenerative process associated with the disease eventually overrides the initial structural advantages observed in females [64].

Sex differences in brain atrophy associated with Alzheimer’s disease (AD) follow stage-dependent trajectories. Women exhibited higher cortical thickness across many regions, whereas men showed steady thinning from normal aging through Alzheimer’s, while women maintained relative stability early on but experienced a more rapid decline at later stages [47]. Female patients experience a more rapid rate of cortical thinning than male patients across all four major lobes of the cortex and earlier hippocampal atrophy, reflecting a faster progression of neurodegenerative changes after disease onset, with the exception of the motor cortex, primary sensory areas, and the inferior frontal lobe [65, 66]. This suggests that sex exerts a stronger influence on the pace of disease progression than on its initial emergence.

Early onset Alzheimer’s Disease patients also have a greater tau pathology when compared to late-onset Alzheimer’s Disease. Differences between the sexes may be more pronounced at higher Aβ levels, where women tend to be more susceptible to early aggregated Aβ secretion of p-tau and may precede positive PET imaging. The interaction between sex and p-tau217 accumulation is even more pronounced in APOE ε4 carriers. It is unclear if this is due primarily to tau accumulation, or also involves inflammatory or synapse biology, which defers between the sexes.

Interestingly, a recent study shows that in Alzheimer’s disease, women have greater pathological burden but better-preserved brain structure than men, including higher cortical thickness and lower white matter damage. This may be partly explained by lower early-life cerebrovascular burden in women, whereas men more frequently develop conditions such as myocardial infarction and stroke at younger ages. Overall, these findings suggest greater brain resilience in women, and these differences were not significantly influenced by age or race [67].

### Anti-amyloid Therapies

Recent advances in disease-modifying therapies in Alzheimer’s disease, such as anti-amyloid immunotherapies lecanemab and donanemab have led to a significant shift in the treatment of AD. However, secondary analyses of anti-amyloid antibody trials have suggested possible sex differences in treatment efficacy. In lecanemab’s phase 3 trial, a supplementary forest plot from their primary result paper indicated a statistically significant sex difference in primary clinical effect [68]. However, the authors note that the study was underpowered for these subgroup analyses. In donanemab’s phase 3 trial, where participants were selected by baseline tangle load, no sex differences were observed [69]. Subsequent renalaysis of lecanemab and donanemab trial data found evidence of lower treatment effect in women than in men for lecanemab, suggesting that the presence of an effect was almost six times more likely than the absence of an effect [70]. For donanemab, there was no evidence of a sex difference in effect [70]. As discussed above, tau accumulates faster in women, which offers a plausible pathway by which faster tangle accumulation could blunt the efficacy of anti-amyloid immunotherapies for women.

### Atypical Variants and Remaining Gaps

Sex-aware interpretation is also relevant in atypical Alzheimer presentations, which often reach general neurology or psychiatry before dementia specialists. Logopenic variant primary progressive aphasia is an instructive example: although often associated with Alzheimer pathology, it is not synonymous with Alzheimer disease, and recent work shows a substantial amyloid-negative subgroup despite similar language impairment profiles. Posterior cortical atrophy has stronger biomarker evidence for underlying Alzheimer disease and appears to affect women somewhat more often, with high rates of amyloid positivity, tau positivity, and autopsy-confirmed Alzheimer pathology. Yet even in posterior cortical atrophy, co-pathologies such as cerebral amyloid angiopathy, Lewy body disease, and cerebrovascular injury remain common and clinically relevant. For lvPPA, dysexecutive Alzheimer disease, and corticobasal syndrome, sex-stratified biomarker trajectories and mixed-pathology frequencies remain inadequately defined. These are important gaps as disease-modifying and biomarker-guided therapies move into more heterogeneous clinic populations [71–73].

### Future Directions

Future research and treatment options, specifically targeting the preclinical phase of Alzheimer’s Disease, may be more efficacious. Targeting tau may be more efficacious in women, given the greater influence tau pathology appears to have in females. One study followed longitudinal plasma p-tau levels and the ratio of phosphorylated to non-phosphorylated p-tau217, which increases throughout the preclinical and early stages of Alzheimer’s Disease. Results demonstrated years until Alzheimer’s Disease onset based on plasma %p-tau217 had a median absolute error of 3–4 years [74]. In addition, a shorter interval of %p-tau217 positivity to symptom onset among older individuals may be helpful specifically in females with comorbidities to help determine appropriate treatment options.

## Conclusion

These findings support a sex-aware approach to assessment. First, verbal list learning should not be used in isolation when women report subjective decline or when family members describe subtle but progressive change. A more balanced battery should include nonverbal or visuospatial memory and executive measures alongside verbal memory. Second, blood biomarkers should be treated as triage tools, not destiny. If plasma p-tau is borderline elevated in a woman with persistent symptoms, preserved verbal memory should not reassure the clinician too quickly; rather, it should lower the threshold for confirmatory CSF or PET testing. Third, tau PET may be particularly helpful in women because it can expose biologic disease that is not yet obvious on routine cognitive screening. Hormonal history can add useful context: female sex, earlier menopause, and later initiation of hormone therapy have each been associated with greater tau vulnerability in the setting of elevated amyloid, supporting more nuanced counseling around midlife history [54, 62, 72, 75].

## Key References

- Coughlan GT, et al. Sex Differences in P-Tau217, Tau Aggregation, and Cognitive Decline. JAMA Neurol. 2026;83:369–381.
  - ○ This study is among the most clinically relevant recent biomarker papers examining sex differences in Alzheimer’s disease. Women demonstrated higher p-tau217 levels and stronger relationships between amyloid burden, tau aggregation, and cognitive decline compared with men. The findings support the concept that women may exhibit greater tau vulnerability despite similar amyloid trajectories and reinforce the emerging role of plasma p-tau217 in early detection and treatment stratification.
- Melville M, et al. Menopause hormone therapy and risk of mild cognitive impairment or dementia: a systematic review and meta-analysis. Lancet Healthy Longev. 2025;6:100803.
  - ○ This systematic review and meta-analysis evaluated the relationship between menopause hormone therapy and later cognitive outcomes. The authors concluded that current evidence does not support a clear protective or harmful effect of menopause hormone therapy on dementia risk overall. This paper is important because it contextualizes the growing interest in estrogen biology and Alzheimer’s disease while emphasizing the limitations of current evidence.
- Tsiknia AA, et al. Sex differences in plasma p-tau181 associations with Alzheimer’s disease biomarkers, cognitive decline, and clinical progression. Mol Psychiatry. 2022;27:4314–4322.
  - ○ This influential study demonstrated that sex modifies the relationship between plasma p-tau181 and downstream Alzheimer’s disease biomarkers, cognitive decline, and clinical progression. Women showed stronger associations between elevated plasma p-tau and neurodegenerative change, suggesting that equivalent biomarker values may carry different clinical implications across sexes.

## Data Availability

No datasets were generated or analysed during the current study.

## References

[1] Women and Alzheimer’s | Alzheimer’s Association. Alzheimer’s Association. https://www.alz.org/alzheimers-dementia/what-is-alzheimers/women-and-alzheimer-s

[2] Ferretti MT, et al. Sex differences in Alzheimer disease - the gateway to precision medicine. Nat Rev Neurol. 2018;14:457–69. 29985474 10.1038/s41582-018-0032-9

[3] Mauvais-Jarvis F, et al. Sex and gender: modifiers of health, disease, and medicine. Lancet. 2020;396:565–82. 32828189 10.1016/S0140-6736(20)31561-0 PMC7440877

[4] Altmann A, Tian L, Henderson VW, Greicius MD. Alzheimer’s Disease Neuroimaging Initiative Investigators. Sex modifies the APOE-related risk of developing Alzheimer disease. Ann Neurol. 2014;75:563–73. 24623176 10.1002/ana.24135 PMC4117990

[5] Ungar L, Altmann A, Greicius MD. Apolipoprotein E, gender, and Alzheimer’s disease: an overlooked, but potent and promising interaction. Brain Imaging Behav. 2014;8:262–73. 24293121 10.1007/s11682-013-9272-x PMC4282773

[6] Rasgon NL, et al. Insulin resistance and hippocampal volume in women at risk for Alzheimer’s disease. Neurobiol Aging. 2011;32:1942–8. 20031276 10.1016/j.neurobiolaging.2009.12.005 PMC2891925

[7] Giuffrè GM, et al. The impact of insulin resistance on cognitive impairment and cerebrospinal fluid biomarkers in Alzheimer’s disease. Neurobiol Dis. 2026;222:107352. 41831578 10.1016/j.nbd.2026.107352

[8] De Paoli M, Zakharia A, Werstuck GH. The Role of Estrogen in Insulin Resistance: A Review of Clinical and Preclinical Data. Am J Pathol. 2021;191:1490–8. 34102108 10.1016/j.ajpath.2021.05.011

[9] Miller EC, et al. Associations between adverse pregnancy outcomes and cognitive impairment and dementia: a systematic review and meta-analysis. Lancet Healthy Longev. 2024;5:100660. 39675366 10.1016/j.lanhl.2024.100660 PMC11726346

[10] Brinton RD, Yao J, Yin F, Mack WJ, Cadenas E. Perimenopause as a neurological transition state. Nat Rev Endocrinol. 2015;11:393–405. 26007613 10.1038/nrendo.2015.82 PMC9934205

[11] Arevalo M-A, Azcoitia I, Garcia-Segura LM. The neuroprotective actions of oestradiol and oestrogen receptors. Nat Rev Neurosci. 2015;16:17–29. 25423896 10.1038/nrn3856

[12] Breeze B, et al. Menopause and Alzheimer’s disease susceptibility: Exploring the potential mechanisms. Brain Res. 2024;1844:149170. 39163895 10.1016/j.brainres.2024.149170

[13] Barth C, Villringer A, Sacher J. Sex hormones affect neurotransmitters and shape the adult female brain during hormonal transition periods. Front Neurosci. 2015;9:37. 25750611 10.3389/fnins.2015.00037 PMC4335177

[14] Melville M, et al. Menopause hormone therapy and risk of mild cognitive impairment or dementia: a systematic review and meta-analysis. Lancet Healthy Longev. 2025;6:100803. 41448220 10.1016/j.lanhl.2025.100803

[15] Maki P, Weber M. Do Menopausal Symptoms Account for the Declines in Cognitive Function During the Menopausal Transition? in 2019;101–9. 10.1007/978-3-030-11355-1_6.

[16] Baker FC, Lampio L, Saaresranta T, Polo-Kantola P. Sleep and Sleep Disorders in the Menopausal Transition. Sleep Med Clin. 2018;13:443–56. 30098758 10.1016/j.jsmc.2018.04.011 PMC6092036

[17] Zeydan B, et al. Sleep quality and cortical amyloid-β deposition in postmenopausal women of the Kronos early estrogen prevention study. NeuroReport. 2021;32:326–31. 33470769 10.1097/WNR.0000000000001592 PMC7878341

[18] Bove R, et al. Age at surgical menopause influences cognitive decline and Alzheimer pathology in older women. Neurology. 2014;82:222–9. 24336141 10.1212/WNL.0000000000000033 PMC3902759

[19] Georgakis MK, Beskou-Kontou T, Theodoridis I, Skalkidou A, Petridou ET. Surgical menopause in association with cognitive function and risk of dementia: A systematic review and meta-analysis. Psychoneuroendocrinology. 2019;106:9–19. 30928686 10.1016/j.psyneuen.2019.03.013

[20] Sáiz-Vázquez O, et al. Depression as a Risk Factor for Alzheimer’s Disease: A Systematic Review of Longitudinal Meta-Analyses. J Clin Med. 2021;10:1809. 33919227 10.3390/jcm10091809 PMC8122638

[21] Brain J, et al. Temporal dynamics in the association between depression and dementia: an umbrella review and meta-analysis. eClinicalMedicine. 2025;84:103266. 40687743 10.1016/j.eclinm.2025.103266 PMC12273843

[22] Elser H, et al. Association of Early-, Middle-, and Late-Life Depression With Incident Dementia in a Danish Cohort. JAMA Neurol. 2023;80:949–58. 37486689 10.1001/jamaneurol.2023.2309 PMC10366950

[23] Wang X, Ye T, Zhou W, Zhang J. Sex-specific association of depressive symptom trajectories with cognitive decline and clinical progression in mild cognitive impairment. Alzheimers Dement. 2025;21:e70548. 40754890 10.1002/alz.70548 PMC12319156

[24] Barth C, Crestol A, de Lange A-MG, Galea LA. M. Sex steroids and the female brain across the lifespan: insights into risk of depression and Alzheimer’s disease. Lancet Diabetes Endocrinol. 2023;11:926–41. 37865102 10.1016/S2213-8587(23)00224-3

[25] Nianogo RA, et al. Risk Factors Associated With Alzheimer Disease and Related Dementias by Sex and Race and Ethnicity in the US. JAMA Neurol. 2022;79:584–91. 35532912 10.1001/jamaneurol.2022.0976 PMC9086930

[26] Wood Alexander M, et al. Cardiovascular contributions to dementia: Examining sex differences and female-specific factors. Alzheimers Dement. 2025;21:e70610. 40851413 10.1002/alz.70610 PMC12375877

[27] Johansson L, et al. Midlife psychological stress and risk of dementia: a 35-year longitudinal population study. Brain J Neurol. 2010;133:2217–24. 10.1093/brain/awq116 20488887

[28] Wallensten J, et al. Stress, depression, and risk of dementia - a cohort study in the total population between 18 and 65 years old in Region Stockholm. Alzheimers Res Ther. 2023;15:161. 37779209 10.1186/s13195-023-01308-4 PMC10544453

[29] Arnsten AFT. Stress signalling pathways that impair prefrontal cortex structure and function. Nat Rev Neurosci. 2009;10:410–22. 19455173 10.1038/nrn2648 PMC2907136

[30] Kwon HS, Koh S-H. Neuroinflammation in neurodegenerative disorders: the roles of microglia and astrocytes. Transl Neurodegener. 2020;9:42. 33239064 10.1186/s40035-020-00221-2 PMC7689983

[31] Sotiropoulos I, et al. Stress acts cumulatively to precipitate Alzheimer’s disease-like tau pathology and cognitive deficits. J Neurosci Off J Soc Neurosci. 2011;31:7840–7. 10.1523/JNEUROSCI.0730-11.2011 PMC6633145 21613497

[32] Cadar D, et al. Individual and Area-Based Socioeconomic Factors Associated With Dementia Incidence in England: Evidence From a 12-Year Follow-up in the English Longitudinal Study of Ageing. JAMA Psychiatry. 2018;75:723–32. 29799983 10.1001/jamapsychiatry.2018.1012 PMC6145673

[33] Cao X, et al. Individual and neighborhood socioeconomic inequality and the risk of dementia: A 14-year follow‐up study. Alzheimers Dement. 2026;22:e71060. 41485131 10.1002/alz.71060 PMC12765403

[34] Ajnakina O, Cadar D, Steptoe A. Interplay between Socioeconomic Markers and Polygenic Predisposition on Timing of Dementia Diagnosis. J Am Geriatr Soc. 2020;68:1529–36. 32187654 10.1111/jgs.16406 PMC7363562

[35] Marden JR, Tchetgen T, Kawachi EJ, I., Glymour MM. Contribution of Socioeconomic Status at 3 Life-Course Periods to Late-Life Memory Function and Decline: Early and Late Predictors of Dementia Risk. Am J Epidemiol. 2017;186:805–14. 28541410 10.1093/aje/kwx155 PMC5859987

[36] Na M, et al. Food Insecurity and Cognitive Function in Middle to Older Adulthood: A Systematic Review. Adv Nutr. 2020;11:667–76. 31711095 10.1093/advances/nmz122 PMC7231583

[37] Wong JC, et al. Food Insecurity Is Associated with Subsequent Cognitive Decline in the Boston Puerto Rican Health Study123. J Nutr. 2016;146:1740–5. 27466603 10.3945/jn.115.228700 PMC4997276

[38] Fisher PJ. Gender and poverty in the United States: Evidence from the Survey of Consumer Finances. PLoS ONE. 2026;21:e0343238. 41811809 10.1371/journal.pone.0343238 PMC12978492

[39] Vervoort D, et al. Addressing the Global Burden of Cardiovascular Disease in Women: JACC State-of-the-Art Review. J Am Coll Cardiol. 2024;83:2690–707. 38897679 10.1016/j.jacc.2024.04.028

[40] Htun HL, et al. Social determinants of health and risk of dementia among older men and women: A 12-year cohort study in Australia. Alzheimers Dement. 2025;21:e70065. 40110677 10.1002/alz.70065 PMC11923569

[41] Bancks MP, et al. Self-reported experiences of discrimination and incident dementia. Alzheimers Dement J Alzheimers Assoc. 2023;19:3119–28. 10.1002/alz.12947 PMC10390651 36724324

[42] Alzheimer’s Association. 2025 Alzheimer’s disease facts and figures. Alzheimers Dement*.* 2025;21:e70235.

[43] Liu M, et al. Sex differences in cognitive performance in Alzheimer’s disease: Insights from the ADAS-Cog-13. J Alzheimers Dis JAD. 2026;13872877261440125. 10.1177/13872877261440125. 10.1177/13872877261440125 41940855

[44] Ryan JJ, Umfleet G, Kreiner L, Fuller DS, A. M., Paolo AM. Neuropsychological differences between men and women with Alzheimer’s disease. Int J Neurosci. 2018;128:342–8. 28926308 10.1080/00207454.2017.1382492

[45] Levine TF, et al. Effect of sex on trajectories of verbal learning and memory and clinical symptoms in diverse Alzheimer’s disease cohorts. J Alzheimers Dis JAD. 2026;13872877261444759. 10.1177/13872877261444759. 10.1177/13872877261444759 42024093

[46] Emrani S, Sundermann EE. Sex/gender differences in the clinical trajectory of Alzheimer’s disease: Insights into diagnosis and cognitive reserve. Front Neuroendocrinol. 2025;77:101184. 39951912 10.1016/j.yfrne.2025.101184 PMC13045642

[47] Cieri F, et al. Relationship of sex differences in cortical thickness and memory among cognitively healthy subjects and individuals with mild cognitive impairment and Alzheimer disease. Alzheimers Res Ther. 2022;14:36. 35193682 10.1186/s13195-022-00973-1 PMC8864917

[48] Sundermann EE, et al. Does the Female Advantage in Verbal Memory Contribute to Underestimating Alzheimer’s Disease Pathology in Women versus Men? J Alzheimers Dis JAD. 2017;56:947–57. 28106548 10.3233/JAD-160716 PMC7644197

[49] Iverson GL, Karr JE. Improving the Methodology for Identifying Mild Cognitive Impairment in Intellectually High-Functioning Adults Using the NIH Toolbox Cognition Battery. Front Psychol. 2021;12:724888. 34566807 10.3389/fpsyg.2021.724888 PMC8457516

[50] Lee S, Kawachi I, Berkman LF, Grodstein F. Education, other socioeconomic indicators, and cognitive function. Am J Epidemiol. 2003;157:712–20. 12697575 10.1093/aje/kwg042

[51] Elkana O, et al. Sensitivity of Neuropsychological Tests to Identify Cognitive Decline in Highly Educated Elderly Individuals: 12 Months Follow up. J Alzheimers Dis JAD. 2016;49:607–16. 26484925 10.3233/JAD-150562

[52] Kang M, et al. Subjective Cognitive Decline Plus and Longitudinal Assessment and Risk for Cognitive Impairment. JAMA Psychiatry. 2024;81:993–1002. 38959008 10.1001/jamapsychiatry.2024.1678 PMC11223054

[53] Boutin S, Houzé B, Pichet Binette A, Brambati SM. & Alzheimer’s Disease Neuroimaging Initiative. Uncovering pathology, subjective cognitive complaints, and sex in early Alzheimer’s disease. J Alzheimers Dis JAD. 2026;109:203–23. 41217839 10.1177/13872877251393268 PMC12722564

[54] Coughlan GT, et al. Sex Differences in P-Tau217, Tau Aggregation, and Cognitive Decline. JAMA Neurol. 2026;83:369–81. 41697669 10.1001/jamaneurol.2025.5670 PMC12910460

[55] Ossenkoppele R, et al. Tau PET positivity in individuals with and without cognitive impairment varies with age, amyloid-β status, APOE genotype and sex. Nat Neurosci. 2025;28:1610–21. 40670684 10.1038/s41593-025-02000-6 PMC12321570

[56] Edwards L, et al. Multimodal neuroimaging of sex differences in cognitively impaired patients on the Alzheimer’s continuum: greater tau-PET retention in females. Neurobiol Aging. 2021;105:86–98. 34049062 10.1016/j.neurobiolaging.2021.04.003 PMC8820163

[57] Banks SJ, et al. Sex differences in Alzheimer’s disease: do differences in tau explain the verbal memory gap? Neurobiol Aging. 2021;107:70–7. 34399127 10.1016/j.neurobiolaging.2021.05.013 PMC8963683

[58] Buckley RF, et al. Sex Differences in the Association of Global Amyloid and Regional Tau Deposition Measured by Positron Emission Tomography in Clinically Normal Older Adults. JAMA Neurol. 2019;76:542–51. 30715078 10.1001/jamaneurol.2018.4693 PMC6515599

[59] Boccalini C, et al. Sex differences in the association of Alzheimer’s disease biomarkers and cognition in a multicenter memory clinic study. Alzheimers Res Ther. 2025;17:46. 39966925 10.1186/s13195-025-01684-z PMC11837373

[60] Kang S, et al. Brain Perfusion, Atrophy, and Dopaminergic Changes in Amyloid Negative Logopenic Primary Progressive Aphasia. Sci Rep. 2025;15:8429. 40069253 10.1038/s41598-025-90116-x PMC11897146

[61] Smith R, et al. The accumulation rate of tau aggregates is higher in females and younger amyloid-positive subjects. Brain J Neurol. 2020;143:3805–15. 10.1093/brain/awaa327 PMC7805812 33439987

[62] Tsiknia AA, et al. Sex differences in plasma p-tau181 associations with Alzheimer’s disease biomarkers, cognitive decline, and clinical progression. Mol Psychiatry. 2022;27:4314–22. 35768637 10.1038/s41380-022-01675-8 PMC9718670

[63] Howe MD, et al. Clinical application of plasma P-tau217 to assess eligibility for amyloid-lowering immunotherapy in memory clinic patients with early Alzheimer’s disease. Alzheimers Res Ther. 2024;16:154. 38971815 10.1186/s13195-024-01521-9 PMC11227160

[64] Sangha O, et al. Structural volume and cortical thickness differences between males and females in cognitively normal, cognitively impaired and Alzheimer’s dementia population. Neurobiol Aging. 2021;106:1–11. 34216846 10.1016/j.neurobiolaging.2021.05.018

[65] Sauty B, Durrleman S. Impact of sex and APOE-ε4 genotype on patterns of regional brain atrophy in Alzheimer’s disease and healthy aging. Front Neurol. 2023;14:1161527. 37333001 10.3389/fneur.2023.1161527 PMC10272760

[66] Inguanzo A, et al. Atrophy trajectories in Alzheimer’s disease: how sex matters. Alzheimers Res Ther. 2025;17:79. 40217302 10.1186/s13195-025-01713-x PMC11987288

[67] Akinci M, et al. Sex Differences in Alzheimer Disease Imaging Biomarkers in a Diverse, Community-Based Cohort. JAMA Netw Open. 2026;9:e2554524. 41591778 10.1001/jamanetworkopen.2025.54524 PMC12848629

[68] van Dyck CH, et al. Lecanemab in Early Alzheimer’s Disease. N Engl J Med. 2023;388:9–21. 36449413 10.1056/NEJMoa2212948

[69] Sims JR, et al. Donanemab in Early Symptomatic Alzheimer Disease: The TRAILBLAZER-ALZ 2 Randomized Clinical Trial. JAMA. 2023;330:512–27. 37459141 10.1001/jama.2023.13239 PMC10352931

[70] Teipel SJ, Tang Y, Khachaturian A. Sex differences in treatment effects of lecanemab and donanemab: A Bayesian reanalysis of CLARITY-AD and TRAILBLAZER-ALZ2. Alzheimers Dement Transl Res Clin Interv. 2025;11:e70155. 10.1002/trc2.70155 PMC12412750 40918062

[71] Taylor B et al. Data-driven neuroanatomical subtypes of primary progressive aphasia. 10.1093/brain/awae314 10.1093/brain/awae314 PMC11884653 39374849

[72] Mielke MM, Fowler NR. Alzheimer disease blood biomarkers: considerations for population-level use. Nat Rev Neurol. 2024;20:495–504. 38862788 10.1038/s41582-024-00989-1 PMC11347965

[73] Chapleau M, et al. Demographic, clinical, biomarker, and neuropathological correlates of posterior cortical atrophy: an international cohort study and individual participant data meta-analysis. Lancet Neurol. 2024;23:168–77. 38267189 10.1016/S1474-4422(23)00414-3 PMC11615965

[74] Petersen KK, et al. Predicting onset of symptomatic Alzheimer’s disease with plasma p-tau217 clocks. Nat Med. 2026;32:1085–94. 41714746 10.1038/s41591-026-04206-y PMC13004683

[75] Sundermann EE et al. Sex-specific norms for verbal memory tests may improve diagnostic accuracy of amnestic MCI. Neurology. 2019;93. 10.1212/WNL.0000000000008467 PMC6946472 31597708

